# Baseline prevalence and correlates of HIV and HCV infection among people who inject drugs accessing a syringe services program; Miami, FL

**DOI:** 10.1186/s12954-020-00385-0

**Published:** 2020-06-10

**Authors:** Tyler S. Bartholomew, Jason Onugha, Corinne Bullock, Carolina Scaramutti, Hardik Patel, David W. Forrest, Daniel J. Feaster, Hansel E. Tookes

**Affiliations:** 1 Department of Public Health Sciences, Miller School of Medicine, University of Miami, 1120 NW 14th St, Miami, FL 33136 USA; 2 Department of Anthropology, College of Arts and Sciences, University of Miami, Miami, FL USA; 3 Department of Infectious Diseases, Miller School of Medicine, University of Miami, Miami, FL USA

## Abstract

**Background:**

Blood-borne viral infections, such as HIV and hepatitis C (HCV), are common infections among people who inject drugs (PWID). This study aims to determine the prevalence of HIV and HCV infection among PWID accessing the first legal syringe services program (SSP) in the state of Florida, along with examining baseline correlates of HIV and HCV infection.

**Methods:**

Baseline behavioral enrollment assessments of 837 participants accessing an SSP for the first time were analyzed. Patients self-reporting or testing HIV or HCV positive at the enrollment visit were included. Socio-demographic, drug use, and injection-related risk behaviors in the last 30 days were compared across groups defined by all combinations of HIV and HCV serostatus.

Bivariate and multivariable logistic regression models were used to assess correlates of baseline HCV and HIV infection independently.

**Results:**

Overall prevalence for HCV and HIV infection were 44.4% and 10.2%, respectively. After adjusting for confounders, the most significant correlates of baseline HCV infection were age (aOR = 1.01), lower education level (aOR = 1.13), currently homeless (aOR = 1.16), injecting more than seven times a day (aOR = 1.14), reusing syringes (aOR = 1.18), and sharing injection equipment (aOR = 1.13). The most significant predictors of baseline HIV infection were age (aOR = 1.01), non-Hispanic Black race (aOR = 1.28), Hispanic ethnicity (aOR = 1.12), gay or bisexual orientation (aOR = 1.22), and methamphetamine injection (aOR = 1.22). In addition, heroin injection (aOR = 0.92) was significantly associated with a lower odds of HIV infection.

**Discussion/conclusion:**

Baseline behavioral predictors differed between HIV infection and HCV infection among participants accessing syringe services. Understanding the risk factors associated with each infection should be considered when developing additional harm reduction interventions tailored for diverse PWID populations served at SSPs.

## Introduction

People who inject drugs (PWID) are at high risk for contracting blood-borne viral infections, including human immunodeficiency virus (HIV) and hepatitis C virus (HCV) [1]. There are approximately 2.3 million people living with HIV with past or present HCV co-infection worldwide, with the majority (1.3 million) cases among people who inject drugs (PWID) [2]. In 2017, approximately 6% of the 38,739 new HIV cases diagnosed in the USA (US) were attributed to PWID [1]. Evidence has shown that PWID living with HIV are six times more likely to be co-infected with HCV than their HIV negative counterparts [2]. In addition, injection drug use remains the leading risk factor for HCV transmission in the US. Between 2010 and 2017, the number of new acute HCV infections reported increased 350%, largely attributed to increased use of injection administration of drugs and development of better surveillance systems [3]. In a systematic review of HIV, HCV, and HBV infection among PWID, an estimated 53.1%, 17.8%, and 9.0% of PWID are HCV antibody, HIV, and HBV surface antigen positive, respectively [4]. However, seropositivity is not a definitive marker for active infection and is unable to differentiate between acute or chronic hepatitis C infection.

In 2017, Miami led the USA in the total number of newly diagnosed HIV cases, with an infection rate of 47 per 100,000 people [1]. In 2017, 11% of people living with HIV in Florida were PWID, and 6% of people who received a new diagnosis reported injection drug use [5]. In addition, the acute HCV infection rate in Florida is almost twice the national average (1.7 per 100,000), highlighting the intersection between injection drug use and blood-borne viral infections in Florida and the need for prevention strategies [3, 5].

However, there remains a major variation in the prevalence of HIV and HCV among PWID across the US [6]. Sharing syringes is the number one route of transmission for HCV and the second riskiest behavior for HIV transmission, after receptive anal sex [3, 7]. Sharing of injection drug preparation equipment, such as cookers and cotton, has been associated with transmission of both HIV and HCV [8, 9]. In the setting of injection drug use, evidence shows that HCV transmission is 5‑25 times more likely than HIV per use of contaminated syringes [10]. This may be related to unique microbiological characteristics that allow HCV to survive on syringes and associated drug paraphernalia longer than HIV [10]. Studies have shown varying HIV and HCV seroprevalence among PWID and identified several risk factors for HIV and HCV including years of injecting, needle and paraphernalia sharing, incarceration, and risky sexual behavior [11–13]. Better understanding of the seroprevalence of blood-borne infections among PWID and greater characterization of risk factors are key components for developing more effective prevention strategies.

Due to increased health risks among PWID, public health interventions and policies have been strategically designed to reduce behaviors that promote the transmission of HIV and HCV in this population. Syringe service programs (SSPs) are harm reduction interventions that are uniquely positioned to mitigate the spread of infectious disease among PWID, and act as a linkage to other healthcare services [14, 15]. Although the implementation of SSPs in the US began in the late 1980s, widespread adoption and implementation has been slow, particularly in the Southern USA [16]. However, southern states have consistently had the highest rates of HIV infection, including Georgia, Louisiana, and Florida, compared to other parts of the country [17]. Historically, primarily in southern urban settings, the predominant drug of choice was crack-cocaine among racial minorities [18], which has been shown to increase HIV risk behaviors [19], HIV treatment disengagement, and viral non-suppression [20]. Not until more recently, due to the opioid crisis, have these communities experienced the increases in injection heroin and methamphetamine use, with southern regions experiencing large increases in methamphetamine-related treatment admissions [21].

Unfortunately, prior to 2016, SSPs were illegal in the State of Florida [22]. Only since July 1, 2019, did SSPs become authorized beyond the pilot program in Miami-Dade County [23]. Since the opening of the IDEA SSP in Miami-Dade County, we have sought to explore HIV and HCV risk factors among PWID accessing our program, a population that has had limited access to harm reduction interventions. This study aims to assess the baseline prevalence and correlates of HIV and HCV infection among PWID accessing SSP services for the first time.

## Methods

### Human subjects

This study was submitted to the Institutional Review Board of the University of Miami (IRB#20190741) and received a determination not to be human subjects research because of the use of anonymous pilot program data. Required collection of pilot data ended after SSPs were authorized statewide in July 2019.

### Participants

Participants included all individuals who enrolled at the IDEA SSP fixed site location. Program participants did not receive any monetary or other types of compensation for enrolling in the SSP or providing their anonymous program data. Data were analyzed on 837 participants who completed an enrollment assessment at the SSP between December 2016 and January 2019. Completion of the survey was not required for enrollment into the SSP, and participants could decline to answer any item.

### Data collection

A 42-item enrollment assessment was developed by the study team, including input from experts at other harm reduction agencies and in coordination with the State of Florida Department of Health for pilot program reporting purposes. Interviewers consisted of IDEA SSP staff who underwent trainings to ensure standardized administration of the assessment. Surveys were administered in a 15-min face-to-face interview in a confidential setting. The interview was conducted while HIV and HCV rapid tests were processing, and basic harm reduction education was delivered post data collection. By statute, no personal identifying information was collected from participants [23]. Survey data were collected and managed on electronic tablets using the REDCap® software [24].

### Measures

#### Socio-demographics

Socio-demographic measures included age (continuous), biological sex (male vs. female), race/ethnicity (non-Hispanic White, non-Hispanic Black, Hispanic), education level (high school education or less vs. education beyond high school), annual income (less than $15,000/year vs. more than $15,000/year), housing status (currently homeless vs. not homeless) and sexual orientation (gay or bisexual vs. heterosexual).

#### Drug use behaviors

Participants were asked to report types of substances injected in the previous 30 days (i.e., heroin, prescription painkillers, cocaine, methamphetamine, crack-cocaine, speedball, or fentanyl/carfentanil).

#### Injection-related behaviors

Participants were assessed for risky injection practices at baseline. Participants reported any sharing of injection equipment (e.g., syringes, cookers, cottons) in the previous 30 days (dichotomized into “shared any” vs. “shared none”). Participants were asked how many times they inject, on average, per day in the previous 30 days (dichotomized into < 7 injections vs. ≥ 7 injections). Reuse of syringes in the previous 30 days (any vs. no reusing) was also assessed.

#### HIV/HCV status

At initial enrollment at the SSP, participants were offered a rapid HIV test via fingerstick using OraQuick Advance® Rapid HIV-1/2 antibody test or Chembio SURE CHECK® HIV 1/2 assay and a rapid HCV test via fingerstick using OraQuick® HCV Rapid antibody test. Prior to biological testing, participants were also asked to self-report current HIV and HCV status. If HIV/HCV antibody testing was declined, the self-reported measure was used for disease status. Both self-reported and biological test data for HIV and HCV were used in the analysis.

#### Statistical analysis

Frequency distributions by HIV and HCV antibody serostatus (HCV mono-infection, HIV mono-infection, HCV/HIV co-infection, and at risk for HIV/HCV infection) were calculated to describe overall sample characteristics. Simple logistic regression models were used to assess the unadjusted associations between socio-demographics, injection drug use, and injection-related behaviors for HCV infection, and HIV, separately. Variables selected in the analysis were based on theoretical hypotheses and existing literature [25–29] on correlates of HCV and HIV infection among this population. All variables selected for the bivariate analyses were used in the final multivariable logistic regression model, and all variables were retained in the final models. Analyses were conducted in M*plus* version 8 [30], and all tests were performed at a significance level of *p* < 0.05.

## Results

### Description of study sample

As of January 2019, there were 837 clients of the SSP that had completed a baseline assessment during initial enrollment into the program. Demographics are presented in Table 1. The average age of participants was 37.7 years old (SD = 10.7). In this sample, the majority of the participants were male (75%), non-Hispanic White (55%) and made less than $15,000/year (53%). In addition, 301 (38%) reported being currently homeless. For HIV and HCV status, 67% and 61% of the baseline HIV and HCV results were confirmed via rapid test, respectively. The overall HCV antibody prevalence among participants was 44.4% (95% CI, 41.0‑48.0) at baseline. The number of participants with reactive HIV tests or history of HIV infection was 10.2% (95% CI, 8.2‑12.4).

**Table 1:** Socio-demographics of HCV mono-infected, HIV mono-infected, HIV/HCV co-infected and PWID with no infection

Variable [*n* (%)]	Total sample (*n* = 837)	HCV mono-infection (*n* = 327)	HIV mono-infection (*n* = 54)	HIV/HCV co-infection (*n* = 27)	No infection (*n* = 390)
Age (mean/SD)	37.7 ± 10.7	38.2 ± 10.0	40.1 ± 10.9	45.8 ± 11.3	34.8 ± 10.4
Gender					
Male	626 (75)	243 (75)	46 (87)	15 (56)	292 (75)
Female	205 (25)	82 (25)	7 (13)	12 (44)	95 (25)
Race/ethnicity					
Non-Hispanic White	445 (55)	180 (57)	17 (31)	10 (37)	217 (58)
Non-Hispanic Black	44 (5)	9 (3)	8 (15)	4 (15)	20 (5)
Hispanic	316 (39)	128 (40)	29 (54)	13 (48)	134 (36)
Sexual orientation					
Straight	685 (83)	291 (89)	9 (17)	21 (78)	333 (87)
Gay/bisexual	139 (17)	36 (11)	44 (83)	6 (22)	49 (13)
Education					
<High school/GED	414 (50)	199 (62)	15 (28)	19 (70)	164 (42)
>High school/GED	408 (50)	124 (38)	38 (72)	8 (30)	222 (58)
Income					
$0‑$14,999	399 (53)	183 (63)	19 (39)	16 (80)	171 (47)
>$15,000	353 (47)	109 (37)	30 (61)	4 (20)	192 (53)
Currently homeless	301 (38)	157 (52)	17 (32)	12 (57)	104 (28)

### Correlates of HCV infection

Results of the bivariate and multivariable logistic regression models for HCV infection are presented in Table 2. Based on the bivariate analyses, age (OR = 1.01; 95% CI, 1.00‑1.01), lower education level (OR = 1.23; 95% CI, 1.15‑1.32), lower income (OR = 1.19; 95% CI, 1.11‑1.28), currently homeless (OR = 1.29; 95% CI, 1.20‑1.39), heroin injection (OR = 1.22; 95% CI, 1.12‑1.33), cocaine injection (1.09, 95% CI, 1.01, 1.18), crack injection (OR = 1.26; 95% CI, 1.12‑1.42), injecting more than seven times a day (OR = 1.22; 95% CI, 1.12‑1.34), reusing syringes (OR = 1.31; 95% CI, 1.16‑1.47), and sharing injection equipment (OR = 1.24; 95% CI, 1.16‑1.33) were significantly associated with higher odds of HCV infection. In addition, participants who reported gay or bisexual orientation (OR = 0.89; 95% CI, 0.81‑0.98), methamphetamine injection (OR = 0.84; 95% CI, 0.76‑0.92), and HIV infection (OR = 0.88; 95% CI, 0.79‑0.99) had significantly lower odds of HCV infection.

**Table 2:** Bivariate and multivariable regression associations between socio-demographics, drug use, and injection risk with HCV infection

Variable	Bivariate associations			Full model		
	OR^a^	95% CI	*P* value	aOR^b^	95% CI	*P* value
*Demographics*						
Age	1.01	1.00‑1.01	< 0.001*	1.01	1.00 1.01	< 0.001*
Race/ethnicity						
Non-Hispanic Black	0.88	0.75‑1.02	0.094	0.89	0.71‑1.10	0.281
Hispanic	1.03	0.96‑1.11	0.342	1.09	0.91‑1.31	0.335
Non-Hispanic White	Ref			Ref		
Biological sex						
Male	0.96	0.88‑1.03	0.264	0.98	0.91‑1.06	0.626
Female	Ref			Ref		
Sexual orientation						
Gay/bisexual	0.89	0.81‑0.98	< 0.001*	0.94	0.35‑2.50	0.211
Straight/heterosexual	Ref			Ref		
Education						
<High school/GED	1.23	1.15‑1.32	< 0.001*	1.13	1.06‑1.21	< 0.001*
>High school/GED	Ref			Ref		
Income						
$0‑$14,999	1.19	1.11‑1.28	< 0.001*	1.05	0.98‑1.13	0.159
> $15,000	Ref			Ref		
Currently homeless	1.29	1.20‑1.39	< 0.001*	1.16	1.08‑1.24	< 0.001*
*Drug use*						
Heroin injection	1.22	1.12‑1.33	< 0.001*	1.05	0.96‑1.14	0.303
Cocaine injection	1.09	1.01‑1.18	0.023*	1.02	0.95‑1.10	0.571
Methamphetamine injection	0.84	0.76‑0.92	< 0.001*	0.94	0.85‑1.05	0.286
Crack injection	1.26	1.12‑1.42	< 0.001*	1.12	0.99‑1.26	0.068
*Injection risk*						
Number of injections per day in the last 30 days						
Greater than 7	1.22	1.12‑1.34	< 0.001*	1.14	1.04‑1.24	0.003*
Less than 7	Ref			Ref		
Reusing syringes						
Any reusing	1.31	1.16‑1.47	< 0.001*	1.18	1.05‑1.32	0.004*
No reusing	Ref			Ref		
Sharing works (syringes, cookers, cottons)						
Shared any works	1.24	1.16‑1.33	< 0.001*	1.13	1.05‑1.21	0.001*
Shared no works	Ref			Ref		
HIV-positive	0.88	0.79‑0.99	0.035*	0.99	0.88‑1.12	0.894

^a^Bivariate logistic regression

^b^Multivariable logistic regression

^*^Significant at alpha < 0.05

In the multivariable analysis, age (aOR = 1.01; 95% CI, 1.00‑1.01), lower education (aOR = 1.13; 95% CI, 1.06‑1.21), currently homeless (aOR = 1.16; 95% CI, 1.08‑1.24), injecting more than seven times a day (aOR = 1.14; 95% CI, 1.04‑1.24), reusing syringes (aOR = 1.18; 95% CI, 1.05‑1.32), and sharing injection equipment (aOR = 1.13; 95% CI, 1.05‑1.21) were significantly associated with a higher odds of HCV infection.

### Correlates of HIV infection

Results of the bivariate and multivariable logistic regression models for HIV infection are presented in Table 3. Based on the bivariate analyses, age (OR = 1.00; 95% CI, 1.00‑1.01), non-Hispanic Black race (OR = 1.24; 95% CI, 1.13‑1.36), Hispanic ethnicity (OR = 1.06; 95% CI, 1.01‑1.10), gay or bisexual orientation (OR = 1.39; 95% CI, 1.32‑1.46), and methamphetamine injection (OR = 1.38; 95% CI, 1.31‑1.46) were significantly associated with higher odds of HIV infection. In addition, heroin injection (OR = 0.80; 95% CI, 0.76‑0.84), reusing syringes (OR = 0.84; 95% CI, 0.78‑0.90), and HCV infection (OR = 0.96; 95% CI, 0.92‑0.99) were significantly associated with lower odds of HIV infection.

**Table 3:** Bivariate and multivariable regression associations between socio-demographics, drug use, and injection risk with HIV infection

Variable	Bivariate associations			Full model		
	OR^a^	95% CI	*P* value	aOR^b^	95% CI	*P* value
*Demographics*						
Age	1.00	1.00‑1.01	< 0.001*	1.00	1.00‑1.01	< 0.001*
Race/ethnicity						
Non-Hispanic Black	1.24	1.13‑1.36	< 0.001*	1.28	1.13‑1.45	< 0.001*
Hispanic	1.06	1.01-1.10	0.013*	1.12	1.02-1.24	0.024*
Non-Hispanic White	Ref			Ref		
Biological sex						
Male	1.01	0.96‑1.06	0.742	0.98	0.94‑1.02	0.315
Female	Ref			Ref		
Sexual orientation						
Gay/bisexual	1.39	1.32‑1.46	< 0.001*	1.22	1.16‑1.29	< 0.001*
Straight/heterosexual	Ref			Ref		
Education						
<High school/GED	0.97	0.93‑1.01	0.151	1.01	0.97‑1.05	0.565
>High school/GED	Ref			Ref		
Income						
$0‑$14,999	1.00	0.95‑1.04	0.820	1.00	0.96‑1.04	0.846
> $15,000	Ref			Ref		
Currently homeless	1.00	0.96‑1.05	0.866	1.02	0.97‑1.06	0.482
*Drug use*						
Heroin injection	0.80	0.76‑0.84	< 0.001*	0.92	0.87‑0.97	0.001*
Cocaine injection	0.96	0.91‑1.01	0.075	0.98	0.94‑1.02	0.422
Methamphetamine injection	1.38	1.31‑1.46	< 0.001*	1.22	1.15‑1.29	< 0.001*
Crack injection	0.95	0.88‑1.02	0.165	0.96	0.89‑1.02	0.178
*Injection risk*						
Number of injections per day in the last 30 days						
Greater than 7	0.95	0.90‑1.01	0.087	0.98	0.93‑1.03	0.438
Less than 7	Ref			Ref		
Reusing syringes						
Any reusing	0.84	0.78‑0.90	0.001*	0.97	0.91‑1.04	0.384
No reusing	Ref			Ref		
Sharing works (syringes, cookers, cottons)						
Shared any works	0.97	0.93‑1.01	0.127	0.99	0.95‑1.03	0.516
Shared no works	Ref			Ref		
HCV-positive	0.96	0.92‑0.99	0.035	1.00	0.96‑1.04	0.894

^a^Bivariate logistic regression

^b^Multivariable logistic regression

^*^Significant at alpha < 0.05

In the multivariable analysis, age (aOR = 1.00; 95% CI, 1.00‑1.01), non-Hispanic Black race (aOR = 1.28; 95% CI, 1.13‑1.45), Hispanic ethnicity (aOR = 1.12; 95% CI, 1.02‑1.24), gay or bisexual orientation (aOR = 1.22; 95% CI, 1.16‑1.29), and methamphetamine injection (aOR = 1.22; 95% CI, 1.15‑1.29) were significantly associated with higher odds of HIV infection. Additionally, heroin injection (aOR = 0.92; 95% CI, 0.87‑0.97) was significantly associated with a lower odds of HIV infection.

## Discussion

This paper presents data from a pilot SSP in a city that previously had no legal harm reduction programs. HIV and HCV prevalence among this sample was low compared to the previously published estimates [4], highlighting potential regional differences in SSP clientele. In the absence of HIV prevention via syringe services, correlates of baseline HCV infection, and baseline HIV infection revealed significantly different risk profiles of PWID. The multivariable analysis is consistent with other studies that show that lower education [25], homelessness [26], frequent injection [27], reuse [28], and sharing of injecting equipment [29], are all associated with higher odds of HCV infection among PWID. These findings suggest that interventions targeting risky injection behavior are essential when opening a new SSP, including evidence-based policies that promote syringe distribution which has been associated with decreased risk of transmission of both HCV and HIV [31–33]. The multivariable analysis also highlights the societal consequences that the social determinants of health, such as limited education and homelessness, have on HCV infection, suggesting that housing may play an important role in HCV prevention among this population. The need for intensive efforts in a variety of high-risk settings to mitigate transmission of HCV via SSPs has been well established [34, 35], including education on the risks of transmission through sharing of injection equipment beyond syringes (i.e., cotton, cooker, water).

Interestingly, in the multivariable analysis, baseline HIV infection was associated with non-Hispanic Black race, Hispanic ethnicity, gay or bisexual orientation, and methamphetamine injection. These findings are consistent with HIV surveillance data in Florida and nationwide which show higher incidence and prevalence of HIV among racial and ethnic minorities [1], a health disparity that should be addressed in a culturally appropriate way when opening a new SSP. In fact, Black race had the highest adjusted odds of baseline HIV infection in our cohort accessing the SSP, supporting the historical intersection between crack-cocaine use and HIV in the south among this racial minority. African Americans comprise 43% of PLWH in Miami, compared to only 18.2% of the county’s population [5]. Nationwide, an escalation in the opioid epidemic has recently occurred in Black communities; opioid deaths have risen 43% among African Americans over the past 5 years compared with a 22% increase among white individuals over the same time period [36]. Correlates of HIV infection also included both gay/bisexual orientation and methamphetamine use. The higher HIV risk among PWID/men who have sex with men is well described in the literature [1, 37]. Our findings suggest that the differing substances that participants are using may play an important role in reducing HIV risk through targeted harm reduction strategies [38]. Conversely, although not significant in the final model, the bivariate analysis showed that men who have sex with men and those who use methamphetamine had a lower risk of HCV infection at baseline. Taken together, our analysis suggests that sexual risk likely plays an important role in HIV infection among sexual minorities and people who injected methamphetamine at the SSP. Methamphetamine injection and disinhibited sexual behavior have been associated with increased risk of HIV [39–42]. The reduced odds of HCV infection paradoxically suggest that men who have sex with men and PWID who injected methamphetamine at our SSP may have had safer injection practices at baseline. In addition, the results suggest that additional HIV prevention strategies, such as pre-exposure prophylaxis (PrEP) and culturally-tailored, evidence-based behavioral interventions, should be targeted toward sexual and ethnic minority PWID who use stimulants. Further investigation into the longitudinal relationships between drugs being injected and HIV or HCV seroconversion could provide important information for potential interventions, including examining risk factors associated with HIV/HCV co-infection.

### Limitations

There are several limitations to this study. First, this study was a survey conducted at a single SSP in one city and may not be generalizable to other cities or to PWID in Miami not using the program. However, it provides an important analysis at the critical opening of an SSP in the city with the highest incidence of HIV in the country [1]. Now that SSP is authorized statewide in Florida this type of information may inform policies and procedures at expansion sites. Another limitation is that self-reported assessments have the potential to introduce social desirability bias. However, trusted SSP staff who have worked in street outreach for years conducted these anonymous assessments in confidential settings, reducing the effect of this potential bias. As we have reviewed and refined policies and procedures now that the pilot period has ended, we have moved to eliminate all unnecessary data collection [43].

Additionally, self-reported HCV and HIV infection was included in the analysis. Twenty-six refused to answer for HCV status and 15 for HIV. Of those enrolled, 234 declined an HCV test and 251 declined HIV testing. Self-report data were combined with participants for whom there was clinical confirmation of status. The rate of decline was high because, during the first years of operation, the SSP staff focused on building relationships, avoiding the perception of any mandatory activities. Finally, it is also important to note that HCV status was determined by antibody or self-report and that chronic infection was not uniformly confirmed via HCV viral load.

## Conclusion

Baseline behavioral predictors differed between HIV infection and HCV infection among participants accessing syringe services for the first time. Understanding the risk factors associated with each infection and subgroups that are disproportionately impacted by HCV and HIV should be considered when developing additional harm reduction interventions that are culturally-tailored for diverse PWID populations seen at SSPs.

## Data Availability

Not applicable

## Abbreviations

PWID: People who inject drugs
HIV: Human immunodeficiency virus
HCV: Hepatitis C virus
HBV: Hepatitis B virus
US: United States
SSP: Syringe service program
IDEA: Infectious Disease Elimination Act
